# Nature-Inspired Molecules as Inhibitors in Drug Discovery

**DOI:** 10.3390/molecules30224372

**Published:** 2025-11-12

**Authors:** Federica Belluti, Alessandra Bisi

**Affiliations:** Department of Pharmacy and Biotechnology, Alma Mater Studiorum—University of Bologna, Via Belmeloro 6, 40126 Bologna, Italy

Natural products (NPs) have historically been regarded as the most important sources of bioactive compounds [1]. Indeed, several clinically approved therapeutics, including antibiotics, anticancer agents, immunosuppressants, and lipid-lowering drugs, have been shown to originate—directly or indirectly—from naturally occurring scaffolds. Their structural diversity, derived from an evolutionary refinement process, has enabled the identification of molecular platforms capable of selectively and effectively interacting with different biological targets [2]. This has prompted the development of synthetic methodologies and medicinal chemistry approaches aimed at modifying these lead compounds to enhance their chemical diversity and improve their biological activity [3].

This Special Issue (SI), titled “Naturally Inspired Molecules as Inhibitors in Drug Discovery”, gathers nine contributions—among which eight are original papers and one is a review article—that exemplify the versatility of NPs in designing inhibitors of many different pathways involved in human diseases. The contributions collected in this SI illustrate how medicinal chemistry approaches and phytochemistry, coupled with pharmacological and computational studies, represent a useful tool in developing new inhibitory therapeutic strategies, covering different areas such as neurodegenerative diseases, viral infections, pain, osteoporosis, and oxidative stress.

One study illustrates the key role of naturally inspired scaffolds in the development of antiviral agents. Benjamin et al. (Contribution 1), by integrating in silico modelling, chemical synthesis, and in vitro validation, presented a series of cannabinoid-inspired inhibitors of SARS-CoV-2 2′-O-methyltransferase enzyme bearing a biphenylpyrane core, among which chromenephenylmethanone-1 (CPM-1) was identified as the most effective and selective inhibitor.

Phytochemical investigations have been exploited by Hamdi et al. (Contribution 2)*,* leading to the discovery of new bioactive metabolites. This detailed study performed on *Asparagus stipularis* revealed the presence of some bioactive components, namely, flavonoids and saponins, endowed with cytotoxic, antioxidant, and lipase-inhibitory effects.

On the other hand, Mukatay et al. (Contribution 3) successfully characterised different secondary metabolites found in the endemic *Artemisia heptapotamica*, thereby emphasising the crucial importance of dereplication strategies in speeding up the identification of new bioactive scaffolds. Remarkably, these studies further strengthened the role of NPs’ biodiversity as a valuable source of molecules and molecular templates endowed with pharmacological effects.

Furthermore, Lisa-Molina et al. (Contribution 4) examined the alkaloid content of autumn-flowering *Narcissus* species to discover new sources of galanthamine, an acetylcholinesterase inhibitor (AChEI) approved for treating Alzheimer’s Disease (AD), as well as to find novel natural AChEIs. These studies exemplify how chemical synthesis and NPs isolation can be exploited in the AD therapeutic context.

Neuropathic pain treatment was addressed in the work of Sałat et al. (Contribution 5), who tested the NPs mirogabalin and cebranopadol in murine models of neuropathy induced by streptozotocin and paclitaxel. Analgesic properties useful in treating this disorder were demonstrated, which is resistant to available treatments. These outcomes further emphasise the translational potential of NPs in clinical settings characterised by inadequate conventional therapies.

From the methanol extract of *Chamaecrista nomame*, Sato et al. (Contribution 6) isolated two classes of cathepsin inhibitors. Cassiaoccidentalin B and torachrysone 8-β-gentiobioside proved to inhibit both cathepsin-K and -B, while pheophytin-*α* only targeted the cathepsin-K isoform. This evidence indicated that pheophytin *a* likely bind to an allosteric site of cathepsin-K and suggested that inhibitors binding to distinct allosteric sites of cathepsin-K may have enhanced inhibitory potency. This approach, relevant to osteoporosis treatment, demonstrates how synergistic inhibition can be leveraged to achieve high specificity while minimising side effects.

Aiello et al. (Contribution 7) designed a set of betalains-inspired analogues bearing a 1,2,3,4-tetrahydropyridine-scaffold, endowed with antioxidant properties. This contribution further demonstrates the prominent role of natural scaffolds as a source of inspiration.

In a further paper, D’Errico et al. (Contribution 8) evaluated the monoamine oxidase (MAO) and xanthine oxidase (XO) inhibitory activity of hydroxytyrosol–donepezil hybrids (HT) previously reported by the same group as neuroprotective compounds in an in vitro neuronal cell model of Alzheimer’s disease. The results led to the identification of inhibitors able to discriminate between the two classes of the selected enzymes involved in both neurodegenerative disorders and oxidative stress. In particular, compounds HT3 and HT4 showed appreciable selective inhibitory activity on the MAO-A isoform; on the other hand, HT2 proved to selectively inhibit XO. This contribution further endorses the effectiveness of the hybridisation strategy involving naturally derived pharmacophoric fragments.

Finally, the review article by Martinez Naya et al. provided a comprehensive overview of cannabidiol (CBD), describing its pharmacokinetic and pharmacological profiles and thus emphasising its multifunctional potential, particularly in therapeutic areas related to inflammation, immunity, and neuroprotection. The increasing significance of phytocannabinoids in translational medicine was also highlighted (Contribution 9).

The articles collected in this SI demonstrate the continuing value of NPs as a source of bioactive lead compounds and as a starting point in medicinal chemistry efforts for the design of compounds acting as inhibitors of diverse pathogenic pathways. By integrating phytochemical research, drug design, synthetic approaches, and pharmacological assessments, the presented contributions demonstrated the interdisciplinary nature of modern drug discovery, while also providing methodological frameworks that may guide future investigations. We would like to thank all the authors for their high-quality contributions and the reviewers for their constructive evaluations, which have ensured the scientific rigour of this collection.
